# Diversity Beyond Insects in Entomology and Allied Disciplines

**DOI:** 10.1093/jisesa/ieaa093

**Published:** 2020-09-15

**Authors:** Alexander B Orfinger

**Affiliations:** 1 Center for Water Resources, Florida A&M University, Tallahassee, FL; 2 Department of Entomology and Nematology, University of Florida, Gainesville, FL

Entomologists study the most diverse group of animals on the planet. Still, a glaring contrast exists when comparing the diversity of the organisms studied with the scientists doing the studying. It is no secret that science fields in the United States, including entomology, suffer from a severe lack of diversity and have since their inception. An internet search of keywords such as ‘persons of color in science’ returns thousands of pertinent results discussing facets including the history, causes, and potential solutions to remedy underrepresentation of Black, Indigenous, and People of Color (BIPOC) communities in the sciences. Searching for ‘persons of color in entomology’ and similar phrases provides dozens to hundreds of relevant results.

Textbooks could revolve around the broader topic of racism in the sciences. This brief letter focuses primarily on one issue in entomology in the United States. As a white man, I do not attempt to discuss the personal experience of BIPOC in entomology, which would be inappropriate and undependable. Instead, I intend to briefly discuss a single, crucial aspect contributing to the overwhelmingly white entomologist workforce: education.

## What is the Problem?

Bottom line: Entomology, as a field, suffers from a lack of diversity that is reflected in higher education. The National Science Foundation found that graduate students in entomology/parasitology in 2016 were 2.3% Black and 4.9% Hispanic/Latinx (National Science Foundation 2016). When compared to the 2016 national population composition of 13.8% Black and 17.3% Hispanic/Latinx (U.S. Census Bureau 2016), it is obvious that these demographics are substantially underrepresented in entomology education. This mismatch has roots in the lack of early exposure to entomology, little sense of belonging in ecology, evolutionary biology, and related disciplines, early education teacher quality, school funding, fewer same-race role models, among other, often interacting, systemic impediments (Abramson et al. 2013, ESA 2015, O’Brien et al. 2020). Once matriculated, BIPOC students may face additional hurdles such as bias in granting decisions and differential treatment from professors and colleagues compared to non-BIPOC peers (Graves Jr 2019), challenging long-term success. A detailed discussion of the myriad barriers that exist is beyond the scope of this letter. Instead, I provide below one example of the historic roots negatively impacting matriculation of racial minorities in agricultural entomology.

### Example: A Roadblock Steeped in History

Data suggest that persons of color can have less vocational interest in agriculture and nature-related fields than white peers (e.g., Kaufman et al. 1998, Outley 2008). So too do most darker-skinned individuals in the United States have American roots stemming from slavery and forced manual labor, often in an agricultural setting. Following slavery, inequities would often manifest as sharecropper subsistence work. These facts are certainly intertwined. The unsavory U.S. origins of many persons of color are a recent memory, with most young people of color having older family members who either worked as stoop laborers or have heard stories of their older relatives doing such work. A narrative was thus born, painting agricultural science and related disciplines as unwelcoming and of low repute (Beck and Swanson 2003). This outlook can serve as a significant cultural deterrent to entering into agricultural science studies, including applied entomology.

## Why Does Diversity in Entomology and Similar Fields Matter?

Aside from the obvious ethical importance of not excluding particular demographics, fostering a more diverse field of entomologists will benefit the science itself. It has been demonstrated that diversity has a strong, positive influence on scientific impact (AlShebli et al. 2018). This fact is important, if not unsurprising. Numerous studies have drawn similar conclusions that diversity of all kinds strongly corresponds to research group performance, collective intelligence, innovation, and quality of science. The entire scientific process from conception to dissemination of results benefits from heterogeneity of perspectives. For example, in the field of population genetics, Gardipee (2007) benefited from her First Nations perspective to develop a noninvasive means of sampling bison DNA during her Masters research. Similarly, the entire subdiscipline of ethnoentomology studies the varied interactions between humans and insects, gleaning knowledge of insect-related medicines, foods, goods, pests, and biocontrol agents from an array of human perspectives (e.g., Dzerefos et al. 2013). The desired diversity of scientific perspectives cannot occur without first cultivating a more diverse cohort of scientists-in-training.

## What Can We Do?

A diverse field of scientists is achievable. A wealth of literature exists discussing strategies to diversify various scientific disciplines (Miriti 2020). Programs, campaigns, and organizations continue to strive to reflect general population demographics. These include Historically Black College and University (HBCU) efforts (e.g., Florida A&M University entomology programs), non-HBCU university efforts (e.g., those by the University of Florida Entomology and Nematology Diversity, Equity, and Inclusion Committee), granting programs (e.g., the NSF INCLUDES initiative), independent organizations (e.g., Entomologists of Color, www.entopoc.org). As scholars, we can use these resources to educate ourselves on why and how to diversify entomology. It is imperative for each of us, particularly those in positions of authority such as tenure-track professors, to work towards alleviating racism. We should perform outreach to diverse student bodies of all ages, mentor BIPOC undergraduates, take on underrepresented graduate students, forge collaborations with BIPOC colleagues, educate ourselves on racial issues, and work to support BIPOC scientists in higher education and beyond. It is not enough to be aware of the problem (as we surely are!). Each of us must actively combat the underlying issues and fight inequality in our field for the betterment of individuals, communities, and the products of our science.

## Closing Thoughts

Evolutionary biologist and science historian Stephen Jay Gould captured a vital and troubling concept when writing ‘I am, somehow, less interested in the weight and convolutions of Einstein’s brain than in the near certainty that people of equal talent have lived and died in cotton fields and sweatshops’. While modern barriers faced by BIPOC to entering into and succeeding in entomology are perhaps less overt in nature, unequally distributed hurdles in society and academia undoubtedly persist. Despite the age of the dialogue surrounding these issues (e.g., Barbosa 1975), representation of BIPOC in life sciences is still woefully low. This letter is not intended to provide any breakthrough solutions. Instead, it is a renewed call to arms. Many potential remedies already exist, yet so does the problem of underrepresentation after so many years of recognition of this critical issue. Entomologists need to have the collective impetus to effect change. As entomologists and as human beings, it is our responsibility to work thoughtfully and diligently to eliminate obstacles and create and maintain a representative workforce for the benefit of the people involved and resulting scientific output.
